# Pediatric Myopia Progression During the COVID-19 Pandemic Home Quarantine and the Risk Factors: A Systematic Review and Meta-Analysis

**DOI:** 10.3389/fpubh.2022.835449

**Published:** 2022-07-22

**Authors:** Ze Yang, Xiang Wang, Shiyi Zhang, Haiyong Ye, Yuanqing Chen, Yongliang Xia

**Affiliations:** ^1^The First Clinical College, Zhejiang Traditional Chinese Medical University, Hangzhou, China; ^2^Department of Orthopedics, Tongde Hospital of Zhejiang Province, Hangzhou, China; ^3^Department of Tuina, The Third Affiliated Hospital of Zhejiang Chinese Medical University, Hangzhou, China; ^4^Department of Internal Traditional Chinese Medicine, The First Affiliated Hospital of Zhejiang Chinese Medical University, Hangzhou, China

**Keywords:** COVID-19, myopia progression, children, risk factors, systematic review and meta-analysis

## Abstract

**Background:**

The COVID-19 pandemic has made many countries adopt restrictive measures like home quarantine. Children were required to study at home, which made parents worried about the rapid myopic progression of their children. To compare myopia progression during the COVID-19 pandemic home quarantine with the time before it and risk factors of myopia progression, we conducted this study.

**Methods:**

We searched PubMed, Embase, the Cochrane Library, and Web of Science to find literature from December 2019 to March 2022 related to COVID-19 pandemic home quarantine and children's myopia progression. Outcomes of myopia progression included axial length and spherical equivalent refraction. Factors of digital screen device time and outdoor activity time were analyzed.

**Results:**

Ten studies were included in this meta-analysis. Compared to the same period before the COVID-19 pandemic, spherical equivalent refraction decreased (OR = −0.27; 95% CI = [−0.33, −0.21]; Z = 8.42; *P* < 0.00001). However, the subgroup analysis showed that there were no significant differences in spherical equivalent refraction between the two groups in higher-grade school-aged children (grades 4 and above, 11 to 18 years old) (OR = 0.01; 95% CI = [−0.05, 0.07]; Z =0.4; *P* = 0.69). The outcome of axial length showed no significant difference (OR = 0.06; 95% CI = [−0.31, 0.44]; Z = 0.34; *P* = 0.74). As for risk factors, the forest plots showed that digital screen device time (OR = 4.56; 95% CI = [4.45, 4.66]; Z = 85.57; *P* < 0.00001) and outdoor activity time (OR = −1.82; 95% CI = [−2.87, −0.76]; Z = 3.37; *P* = 0.0008) were risk factors of myopia progression.

**Conclusion:**

Compared with the time before the COVID-19 pandemic, myopia progression in children during COVID-19 pandemic home quarantine was accelerated, especially in younger children. Increased digital screen device and decreased outdoor activity times were risk factors. When home quarantine eases, more time on outdoor activities and less time on digital screen devices are needed for children.

**Systematic Review Registration:**

https://www.crd.york.ac.uk/prospero/logout.php.

## Introduction

The novel coronavirus disease 2019 has become a worldwide pandemic within several months since December 2019 when it first emerged (1, 2). In March 2020, the WHO declared COVID-19 a global pandemic, which made many countries adopt some important restrictive measures on mobility (3). Home quarantine was required to prevent the spread of the infection. Face-to-face education was interrupted, and online study at home using digital screen devices was started (4, 5). Children were increasingly exposed to digital devices at a close working distance, such as smartphones, tablets, and computers. Moreover, their outdoor activity times were decreased (6). Only understanding the factors associated with this phenomenon can we take measures and prevent it. A study has shown that usage of digital devices was associated with myopia (7). Children who spend more time outdoors have a lower incidence of myopia (8–10).

The main objective of this study was to compare myopia progression in children before the COVID-19 pandemic and during COVID-19 pandemic home quarantine and at the same time explore the risk factors of myopia progression.

## Materials and Methods

### Data Source

PubMed, Embase, the Cochrane Library, and Web of Science were searched in English using the following search terms: (Myopia[Mesh] OR Nearsightedness [Title/Abstract] AND (COVID-19[Mesh] OR COVID-19 Virus Disease [Title/Abstract] OR COVID-19 Virus Infection [Title/Abstract] OR 2019-nCoV Infection [Title/Abstract] OR Coronavirus Disease-19 [Title/Abstract] OR 2019 Novel Coronavirus Disease [Title/Abstract] OR 2019-nCoV Disease [Title/Abstract] OR SARS Coronavirus 2 Infection [Title/Abstract] OR SARS-CoV-2 Infection [Title/Abstract] OR (COVID-19 Pandemic [Title/Abstract]) AND (Child[Mesh] OR Children [Title/Abstract] OR Adolescent [Title/Abstract] OR teenager [Title/Abstract]). We searched them for updated articles published from the inception of each database to 1 March 2022. References of related articles were also searched for other relevant potential studies to ensure that no research studies were overlooked.

### Inclusion and Exclusion Criteria

Our meta-analysis has been reported in conformity with the Preferred Reporting Items for Systematic Reviews and Meta-Analyses (PRISMA) Statement and has been registered at International Prospective Register of Systematic Reviews (number: CRD42021293405) (11). Inclusion criteria were as follows: (1) the aim of the primary studies: comparing myopia progression during pandemic home quarantine with the period before it; (2) children and adolescents aged < 19 years old. Exclusion criteria were those with systemic diseases or with present eye diseases or injuries. Besides, abstracts, reviews, case reports, letters, duplicate publications, or studies with incomplete or unidentified data were also excluded.

### Quality Assessment and Data Extraction

The checklist recommended by the Agency for Healthcare Research and Quality (AHRQ) (12) was used to evaluate the quality of all studies by two independent investigators (Z.Y. and X.W.). The two investigators (Z.Y. and X.W.) read the title, abstract, and full text, screened the literature according to the inclusion and exclusion criteria, and cross-checked the results. If there is a disagreement, the third researcher (Y.L.X.) will be consulted. The extracted data included first author, publication time, source of population, mean age or age range, sample size, outcomes, and risk factors.

### Statistical Analysis

The dichotomous data were expressed as pooled odds ratio (OR) and 95% confidence intervals (95% CI); for continuous outcomes, and the standard (std) mean difference was calculated with mean difference (MD) and 95% CI. A random-effects model was used for the meta-analysis in this study. Statistical heterogeneity was considered present when *p* < 0.1 or I^2^ > 50%. When there was high heterogeneity, a sensitivity analysis was conducted to analyze it. Publication bias was evaluated visually by funnel plots when the inclusion was more than 10 articles. *P* < 0.05 was considered statistically significant. All the statistical analyses were performed with the Revman 5.4 software.

## Results

### Search Results

**Figure 1:**
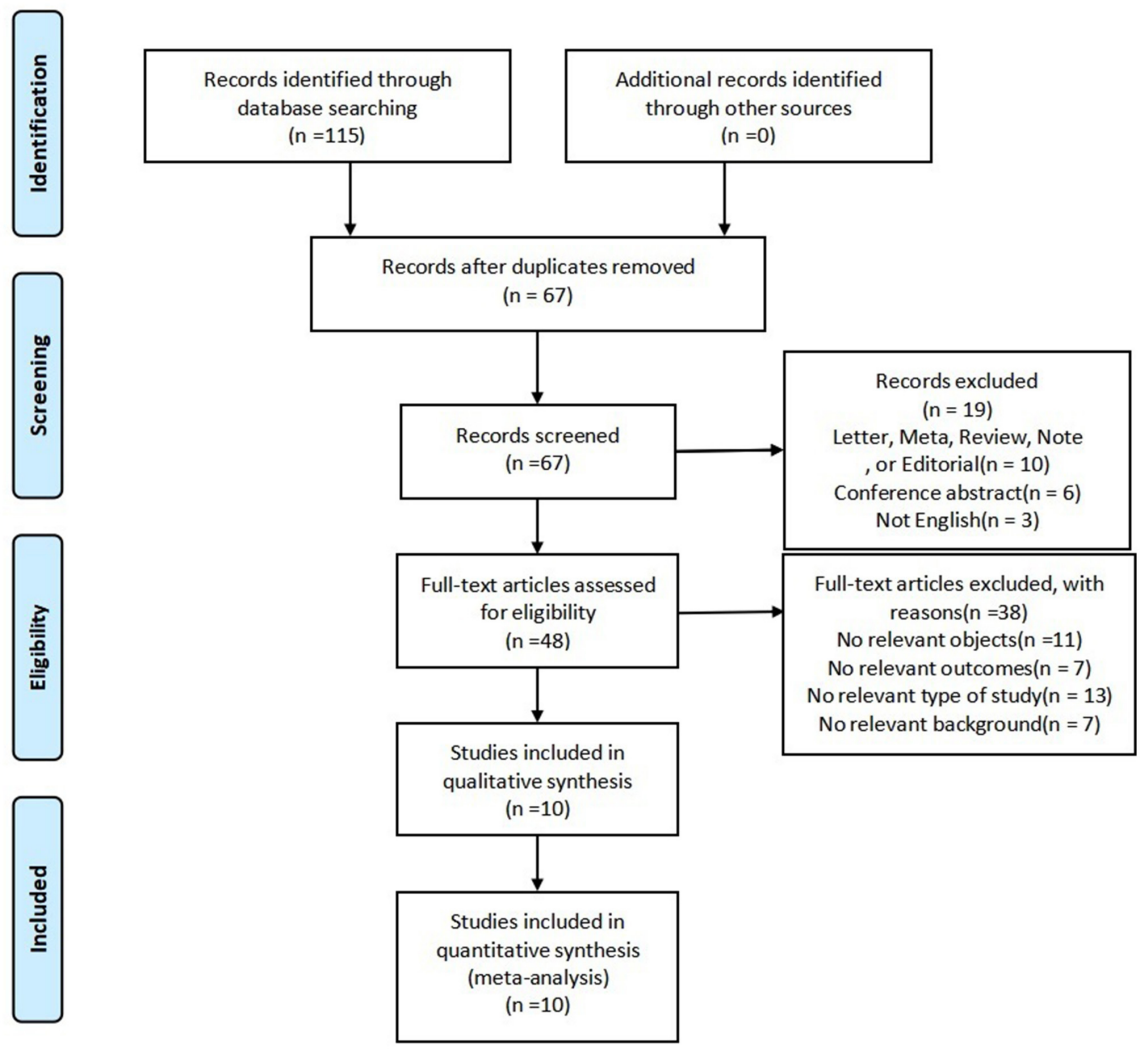
PRISMA flow diagram of the study selection process.

A total of 115 relevant articles were searched according to our search strategy, 48 articles were removed because of duplication, and 67 were screened. After screening the titles and abstracts, 19 studies were excluded. Forty-eight articles were reviewed, among which 11 had no relevant objects, 7 had no relevant outcomes, 13 had study types that were not relevant, and 7 had no relevant background. Eventually, a total of 10 eligible articles were included in this meta-analysis (refer to Figure 1 for details).

### Study Characteristic

A total of 404,177 cases in the ten studies (13–22) were included in this meta-analysis. Of the included studies, six were from China, two were from Turkey, one was from Spain, and one was from Korea. All of them are cross-sectional studies. The outcomes of this meta-analysis were shown as follows: axial length and spherical equivalent refraction. Digital screen device time and outdoor activity time were risk factors. The main characteristics of the included 10 articles are demonstrated in Table 1. AHRQ scores suggested that all the studies scored eight and were all of high quality.

**Table 1:** Characteristics of the 10 studies included in the meta-analysis.

**Study**	**Source of population**	**Mean age or age range (AVG)**	**Sample size (boys/girls)**	**Outcomes**	**Risk factors**	**Quality score**
Alvarez et al. (13)	Spain	5–7	5827(2972/2855)	②	/	8
Aslan et al. (14)	Turkey	12.06 ± 2.29	115 (40/75)	②	/	8
Hu et al. (15)	China	Grade 2 to Grade 3	2114	①②	/	8
Ma et al. (16)	China	8.9 ± 0.69	208(109/99)	①②	③④	8
Ma et al. (17)	China	9.9 ± 1.7	201	②	③④	8
Ozturk et al. (18)	Turkey	13.15 ± 2.03	64(34/30)	①②	/	8
Wang et al. (19)	China	6–13	123535(64335 /59200)	②	/	8
Wang et al. (20)	China	Grade 1 in primary school to Grade 2 in high school	3461(1720/1741)	②	/	8
Yum et al. (21)	Korea	10.1 ± 2.5	103(45/58)	②	④	8
Zhang et al. (22)	China	6-8	1793	①②	③④	8

*①Axial length ②Spherical equivalent refraction ③Digital screen devices time ④Outdoor activities time*.

### Axial Length

**Figure 2:**
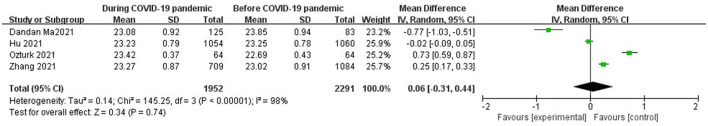
Forest plots for comparison of axial length between COVID-19 pandemic and before it.

As shown in Figure 2, four related studies (15, 16, 18, 22) adopted a random-effects model for the pool of data. The meta-analysis indicated there had no significant difference between two groups in axial length (OR = 0.06; 95% CI = [−0.31, 0.44]; Z = 0.34, *P* = 0.74).

### Spherical Equivalent Refraction

**Figure 3:**
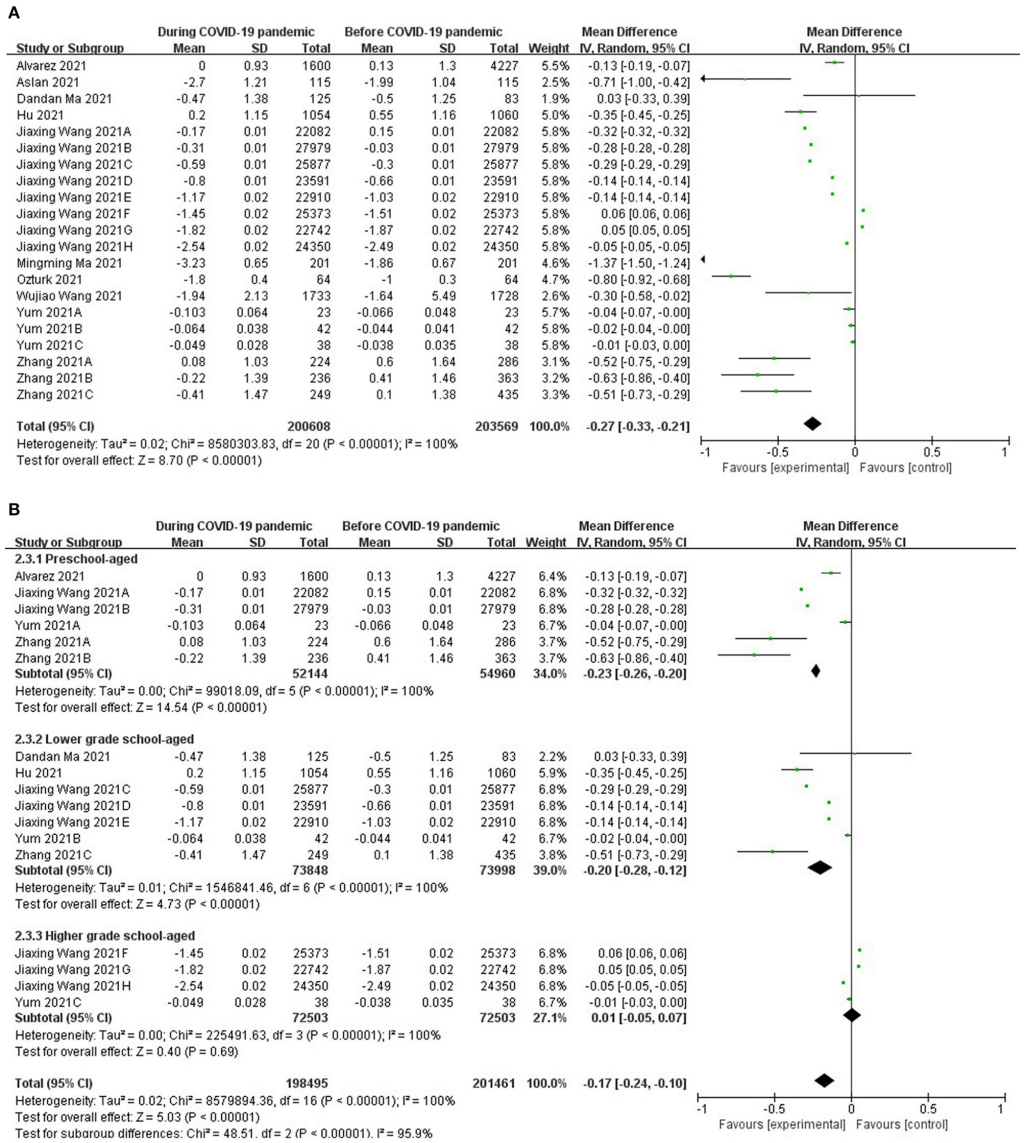
**(A)** Forest plots for comparison of spherical equivalent refraction between COVID-19 pandemic and before it. **(B)** Forest plots and subgroup analysis for comparison of spherical equivalent refraction between COVID-19 pandemic and before it.

Data from the ten related studies (13–22) with this outcome are synthesized. The forest plots showed that there was a significant difference between two groups in spherical equivalent refraction (OR = −0.27; 95% CI = [−0.33, −0.21]; Z = 8.42; *P* < 0.00001) (Figure 3A). The heterogeneity test analysis suggested there was high heterogeneity (I^2^ = 100%, *P* < 0.00001), so the random-effects model was used.

The subgroup analysis showed that there were significant differences in spherical equivalent refraction between two groups of children who were preschool-aged (5 to 7 years old) (OR = −0.23; 95% CI = [−0.26, −0.2]; Z = 14.54; *P* < 0.00001), and there were also significant differences in children who were lower-grade school-aged (grades 1 to 3; 8 to 10 years old) (OR = −0.2; 95% CI= [−0.28, −0.12]; Z = 4.73; *P* < 0.00001). However, there was no significant difference in children who were higher grade school-aged (grades 4 and above, 11 to 18 years old) [OR = −0.01; 95% CI= (−0.05, 0.07); Z =0.4; *P* = 0.69] (Figure 3B).

### Risk Factors of Digital Screen Device Time

**Figure 4:**
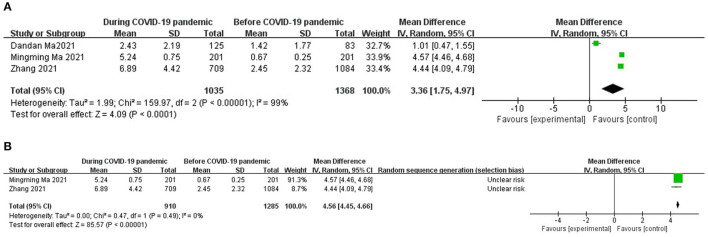
**(A)** Forest plots for comparison of digital screen devices time between COVID-19 pandemic and before it. **(B)** Sensitivity analysis for comparison of digital screen devices time between COVID-19 pandemic and before it.

As for risk factors, three studies (16, 17, 22) were included in the factors of digital screen device time. There was high heterogeneity (I^2^ = 99%, *P* < 0.00001) (Figure 4A).

The sensitivity analysis of the data suggested that the data of Dandan Ma's research were the main source of heterogeneity in the digital screen device time group; after removing that research, the heterogeneity disappeared (I^2^ = 0%, *P* = 0.49) (Figure 4B). The meta-analysis demonstrated that digital screen device time was a risk factor of myopia (OR = 4.56; 95% CI = [4.45, 4.66], Z = 85.57, *P* < 0.00001).

### Risk Factors of Outdoor Activity Time

**Figure 5:**
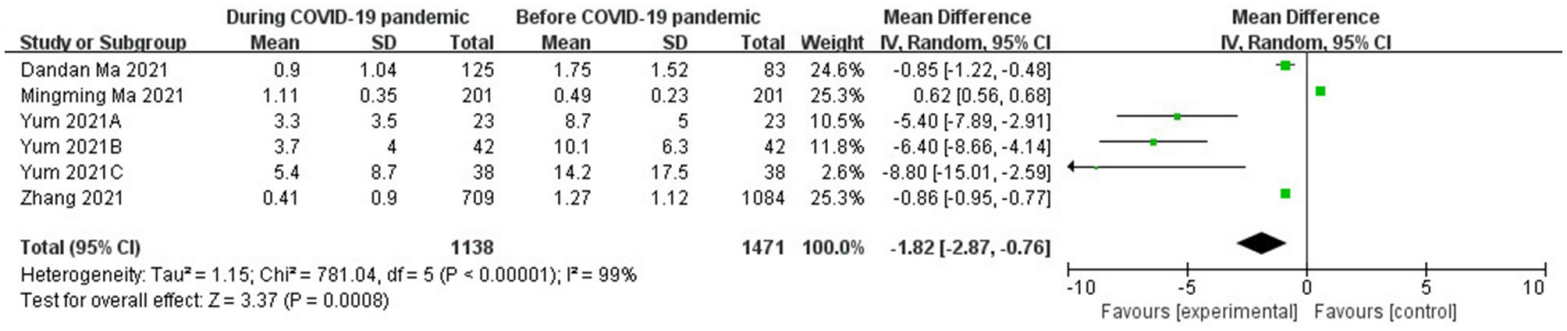
Forest plots for comparison of outdoor activities time between COVID-19 pandemic and before it.

The meta-analysis of data from 1,138 cases during the pandemic and 1,471 cases before the pandemic in four studies (16, 17, 21, 22) indicated that outdoor activity time had significant effects on myopia (OR = −1.82; 95% CI = [−2.87, −0.76]; Z = 3.37; *P* = 0.0008) (Figure 5). High heterogeneity existed (I^2^ = 99%, *P* < 0.00001), so the random-effects model was used.

### Publication Bias

**Figure 6:**
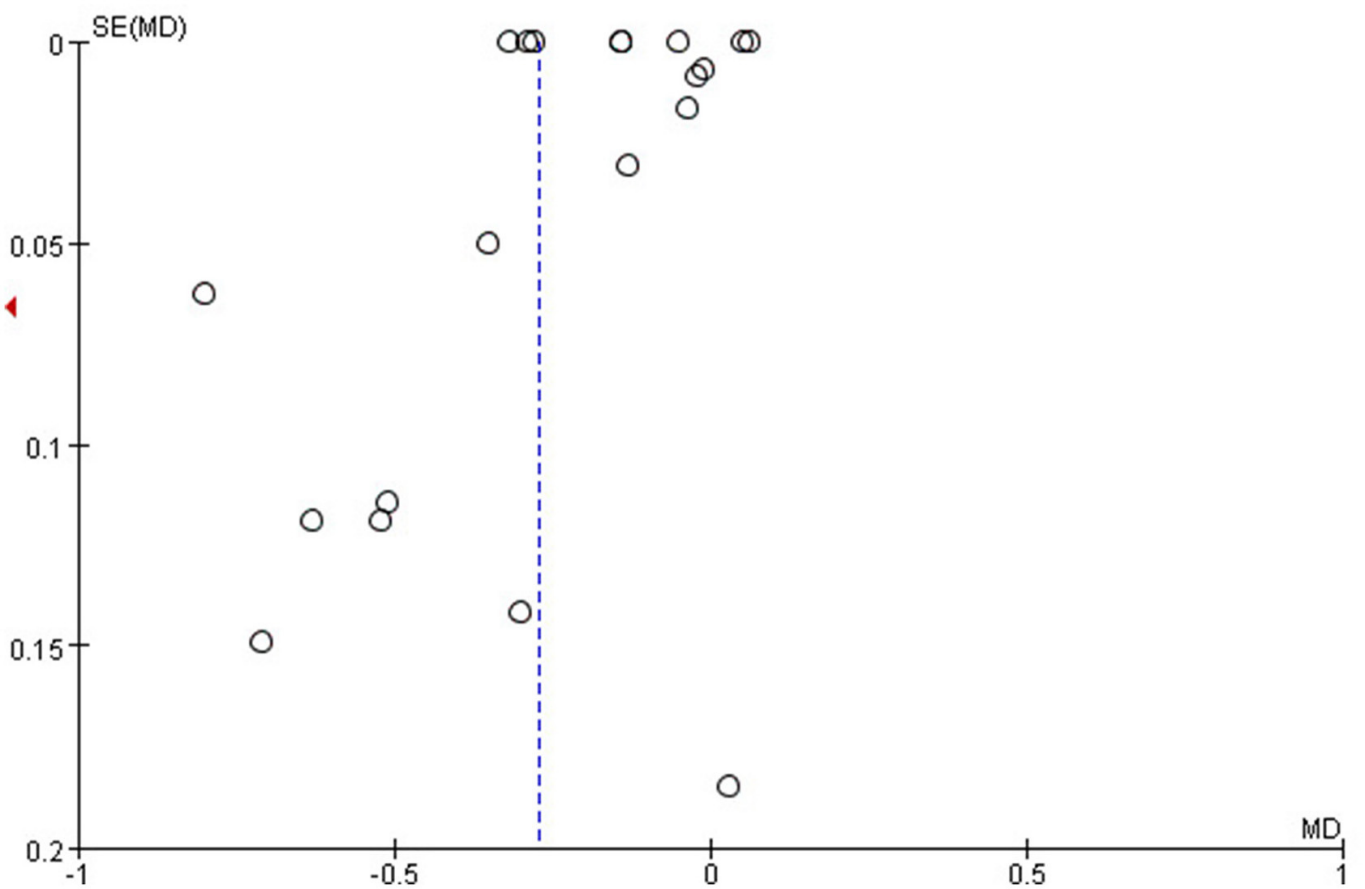
The plot of the spherical equivalent refraction during COVID-19 pandemic and before it.

The funnel plots were generated by Revman 5.4 to test for publication bias. According to the plots (Figure 6), the asymmetry indicated that potential publication bias might influence the results of this review. However, because of insufficient data from the few included studies, the authors were unable to adjust for publication bias. Bias might result from these reasons: the studies included in this meta-analysis were observational, the data were from different countries, and the sample size of the studies ranged differently, which are susceptible to publication bias.

## Discussion

Myopia has emerged as a significant public health problem (23). The World Health Organization (WHO) estimates that half of the population of the world may be myopic by 2050 (19). During the COVID-19 pandemic, countries like China, Turkey, Spain, and South Korea have adopted different measures to prevent the spread of the infection. The measures included home confinement and school closure, and online e-classes were started. More detailed measures were adopted in some countries, i.e., in Spain, it made a transition plan which was divided into four phases by which the mobility of citizens was expanded (13). In China, the limitation on outdoor activity was relaxed after the spread of COVID-19 was effectively contained, and some children had a small increase in outdoor time, whereas most students still continued to study online(17), inevitably leading to children consuming excessive time on digital screen devices and less time on outdoor activities (24, 25). It was called “substitution effect,” which meant that the decrease in time spent on outdoor activities was associated with the increase in time spent on digital screen devices (26). All in all, all these measures caused myopia progression in children.

This review has presented that the home quarantine for COVID-19 has effects on myopic progression among children with a decrease in mean spherical equivalent refraction. The forest plots showed increase in digital screen device time and decrease in outdoor time were risk factors for myopic progression during COVID-19 pandemic home quarantine. Other studies also reported this conclusion. He et al. (8) have shown in their study that myopia incidence was 23% less in children with an additional 40 min of outside activity. Aslan et al. (14) have reported that myopia progression was decreased by 33% in children with 2 h of outdoor activity daily. Ma et al. (16) have reported that digital screen device time was a risk factor for increasing myopia progression. Harrington et al. (27) reported that time spent on digital screen devices was related to increase in myopia prevalence, especially when digital screen time is more than 3 h/day. Different digital screen devices had different effects on myopia progression. Ma et al. (17) reported that myopia progression was slower when using long-distance digital devices such as projectors and televisions. It was because the distance was always < 50 cm when using digital screen devices like smartphones or tablets. Shorter viewing distances require increased accommodation effort, which would increase the progression of myopia.

The subgroup analysis showed that there were significant differences in spherical equivalent refraction between the two groups of children who were preschool-aged (5 to 7 years old) and lower-grade school-aged (grades 1 to 3; 8 to 10 years old). However, there was no significant difference in children who were higher-grade school-aged (grades 4 and above, 11 to 18 years old). It showed the decreasing of spherical equivalent refraction in older children were less obvious than that in younger children, but it didn't mean there was no decreasing in older children' spherical equivalent refraction. It might be because lifestyle changes in older children were not as obvious as those in young children, for they have already been exposed to digital screen devices for a long time. Another possibility was that younger children's refractive status might be more sensitive to lifestyle changes taken by home quarantine during the pandemic than older ages, for they were in a critical stage for the development of myopia (19).

According to this meta-analysis, there was no significant change in axial length in the two groups. Ma et al. (16) suggested that it may be because the myopia in COVID-19 pandemic home quarantine, which is caused by accommodative spasm, was transient. Hu et al. (15) reported that the axial length during the pandemic was shorter than it was before the pandemic, but axial length elongation during periods before pandemic was longer. Actually, axial length was increasing rapidly during the pandemic, but axial length elongation was not reported in other studies; thus, we have to include axial length as an outcome. Besides, a number of articles reported that the outcome for axial length and sample size was small, which would lead to this result as well.

Some other outcomes were also reported by studies. Wang et al. (20) reported that the mean uncorrected visual acuity (UCVA) during the COVID-19 pandemic was higher than it was before the pandemic. Ma et al. reported that there was no significant change in UCVA between two groups. They speculated that it may be transient myopia caused by accommodative spasm (16).

Even though without home quarantine in COVID-19 pandemic, the prevalence of myopia will grow continuously following the prediction made by Holden et al. (28), the great change in lifestyle during COVID-19 pandemic will prompt the change quickly achieved than expected. Because it is a warning to policymakers, parents, and people related to it. Efforts should be taken to prevent the worsening of such progression. Outdoor activity times should be increased for children to keep a healthier lifestyle during the pandemic on the premise of sufficient social distance. Cities in China have already taken the measure of increasing outdoor time as a way to prevent myopia (29, 30). Time spent on digital screen devices should be decreased; if necessary, use long-distance devices such as projectors and televisions instead.

This review has several limitations. First, the sample size of this meta-analysis was small. Because of the limited number of included studies, we were unable to conduct subgroup analysis by countries. Second, research studies on the COVID-19 pandemic are updated rapidly, which may impact the findings. Third, the studies included in this meta-analysis were all observational ones, the data were from different countries and the sample size of the studies ranged differently (from 64 to 123,535), which caused publication bias and high heterogeneity. More data and studies are needed to support this hypothesis in the future.

## Conclusions

In conclusion, compared with time before the COVID-19 pandemic, myopia progression in children during COVID-19 pandemic home quarantine was accelerated. Only by understanding the factors leading to this phenomenon can we prevent it. The increase in digital screen device time and the decrease in outdoor activity time during the COVID-19 pandemic aggravated myopia progression. Policymakers, eye care professionals, educators, and parents should pay more attention to this phenomenon. When home quarantine eases, more time is needed on outdoor activities and less time on digital screen devices for children's schedule.

## Data Availability Statement

The original contributions presented in the study are included in the article/supplementary material, further inquiries can be directed to the corresponding author.

## Author Contributions

Data curation and visualization: ZY, XW, SZ, and YC. Formal analysis and writing—original draft: ZY and XW. Investigation: ZY, XW, and YX. Methodology: ZY, XW, SZ, and HY. Project administration: ZY. Resources: XW and YX. Software: ZY, XW, and HY. Supervision: ZY and HY. Validation: ZY and YX. Writing—review and editing: YC and YX. All the authors have read and agreed to the published version of the manuscript.

## Conflict of Interest

The authors declare that the research was conducted in the absence of any commercial or financial relationships that could be construed as a potential conflict of interest.

## Publisher's Note

All claims expressed in this article are solely those of the authors and do not necessarily represent those of their affiliated organizations, or those of the publisher, the editors and the reviewers. Any product that may be evaluated in this article, or claim that may be made by its manufacturer, is not guaranteed or endorsed by the publisher.
